# Structural Inequalities in Ambulatory Care: A Scoping Review of Access and Quality and the Neglected Dimension of Patient Safety

**DOI:** 10.3390/ijerph23081021

**Published:** 2026-08-04

**Authors:** Andreas Müller, Eitan Bronschtein, Ferdinand Sasváry

**Affiliations:** Department of Public Health, St. Elizabeth University of Health and Social Work, 811 02 Bratislava, Slovakia; ebronschtein@gmail.com (E.B.); sasvary@vssvalzbety.sk (F.S.)

**Keywords:** structural inequality, health equity, ambulatory care, patient safety, healthcare disparities

## Abstract

**Highlights:**

**Public health relevance—How does this work relate to a public health issue?**

- Ambulatory care is where most people meet the health system, so structural inequalities embedded in its organisation shape who obtains timely, high-quality and safe care.
- Inequitable ambulatory care contributes to avoidable illness, delayed diagnosis and preventable harm that fall disproportionately on socially disadvantaged populations.

**Public health significance—Why is this work of significance to public health?**

- This is, to our knowledge, the first scoping review to map structural inequality across access, quality and patient safety together, revealing that safety is by far the least-studied of the three domains.
- Across geographic, financial, linguistic and institutional determinants, structural disadvantage operates through convergent mechanisms, indicating that safety failures in ambulatory care are systematic rather than isolated incidents.

**Public health implications—What are the key implications or messages for practitioners, policy makers and/or researchers in public health?**

- Practitioners and policy makers should treat patient safety as an equity outcome in its own right, with harm surveillance stratified by structural disadvantage.
- Researchers should prioritise safety studies in non-USA and low- and middle-income settings and target system-level vulnerabilities rather than individual provider behaviour.

**Abstract:**

(1) Background: Ambulatory services are the main interface between populations and health systems, yet their benefits are unevenly distributed due to structural inequalities—systemic differences rooted in the social, economic, and political organisation of society. Inequities in access and quality are well documented, but patient safety remains the least examined. (2) Methods: Following Joanna Briggs Institute methodology and PRISMA-ScR guidelines, PubMed/MEDLINE and Scopus were searched for studies published from 2010 to 2026. In total, 44 studies were included, predominantly from the USA (*n* = 31), and synthesised narratively. (3) Results: Inequalities operated through geographic maldistribution, financial barriers, exclusionary institutional cultures, and market-driven practices. Access and quality were each addressed by 17 studies, whereas only four examined patient safety directly; the limited safety evidence indicated disproportionate diagnostic errors, medication-safety failures, and care-coordination breakdowns among vulnerable groups. Evidence was concentrated in the USA. (4) Conclusions: Structural inequalities are well documented for access and quality but are markedly under-studied for patient safety—the principal evidence gap identified. Patient safety warrants treatment as an equity outcome, measured with stratification by structural disadvantage and addressed through system-level interventions.

## 1. Introduction

Ambulatory health services are the most basic point of contact between populations and health systems worldwide, including in low- and middle-income countries. They encompass primary care, outpatient specialist services, community health services, and emergency department care without admission [1]. Ambulatory care is the principal entry point for prevention, diagnosis, treatment, and the management of chronic conditions, and in most OECD countries it accounts for the bulk of all healthcare encounters [2]. Despite this central role, the benefits of ambulatory care are not equally distributed. Its delivery is shaped by structural inequalities, defined as systemic differences in health and healthcare that arise from the social, economic, and political organisation of society. These inequalities produce divergent experiences of access, quality, and safety for disadvantaged populations [3].

Throughout this review, structural inequality denotes the systemic, institutionally embedded production of disadvantage; structural inequity denotes its avoidable and unjust dimension; and healthcare disparity denotes the resulting measurable differences in access, quality, or safety between groups. Structural inequality shifts the analytic focus from individual-level disparity to the institutional arrangements, policies, and historical patterns of resource allocation that generate and sustain disadvantage for particular groups [4]. In ambulatory care, these forces are expressed through geographic inequity in facilities and providers, insurance and payment structures, organisational practices that disadvantage patients with complex social needs, and institutional cultures that fail to accommodate diverse linguistic, cultural, or disability-related requirements [5]. Such factors operate independently of individual patient attributes yet exert a powerful influence on the availability, quality, and safety of care across population groups [6].

These inequalities are not confined to a single point of care; they accumulate across successive encounters and persist over time. Barriers to preventive and primary care delay the diagnosis of chronic conditions and worsen disease control, which raises the risk of avoidable hospitalisation at substantial human and economic cost [7]. Differences in provisioning, provider training, and organisational capacity then widen these outcome gaps across the life course [8]. The same structural pressures may also extend to patient safety: vulnerable populations, often lacking the health literacy or social capital required to navigate complex systems, are reported to be disproportionately affected by medication errors, diagnostic delays, and failures of care coordination [9]. A systematic review of primary care confirms that these safety events fall disproportionately on women and ethnic minority patients [10], while an OECD analysis attributes roughly half of the global burden of patient harm to primary and ambulatory settings, where patients with complex social needs are most exposed [11]. Yet, while inequities in access and quality are comparatively well documented, the evidence on patient safety as a structurally patterned outcome remains notably sparse. This contrast motivates the present review, which foregrounds patient safety as a critical but under-researched dimension of equity in ambulatory care. This dimension warrants explicit characterisation alongside the more extensively studied domains of access and quality.

Recent global developments have sharpened the need to address these inequalities. Service disruptions during the COVID-19 pandemic exposed and amplified existing disadvantage among marginalised communities [12]. The rapid expansion of telemedicine, while promising, introduced new forms of digital exclusion for populations with limited digital literacy or unstable connectivity [13]. Extreme weather events are increasingly recognised as structural determinants of ambulatory care delivery, with disadvantaged populations most exposed to their effects [14].

The evidence base nonetheless remains fragmented across disciplines, settings, and geographic contexts [15]. Previous reviews have typically mapped a single dimension of disadvantage, such as racial, rural–urban, or socioeconomic, without integrating the structural influences that act simultaneously on access, quality, and safety [16]. Most of the literature originates in the USA, where a fragmented and complex insurance landscape generates distinctive structural problems whose findings do not readily generalise to the more integrated financing systems of most European countries [17].

The aim of this scoping review is to systematically map the breadth, range and nature of the evidence on structural inequalities in ambulatory care and their implications for access, quality, and patient safety. The review adopts a conceptualisation of structural inequality that integrates socioeconomic, geographic, demographic, and institutional dimensions [18], and covers literature published between 2010 and 2026—a period in which health equity rose to prominence on policy agendas worldwide [19]. Specifically, the review sought to characterise the structural inequalities documented in ambulatory care, describe their impact on access, quality, and safety outcomes, assess the relative volume and nature of the evidence in each domain, and identify gaps in the evidence base, particularly regarding patient safety, to inform future research priorities.

## 2. Materials and Methods

This scoping review followed the Joanna Briggs Institute (JBI) methodology for scoping reviews and the Preferred Reporting Items for Systematic Reviews and Meta-Analyses extension for Scoping Reviews (PRISMA-ScR) [20,21]. A scoping review design was selected because the objective was to map the extent, range, and nature of the evidence rather than to synthesise effect estimates [22,23]. The synthesis is narrative but rests on a systematic, reproducible search. It gives particular attention to the uneven representation of the three domains (access, quality, and patient safety) in the evidence, while remaining descriptive rather than thesis-driven and making no causal claims. The review was not registered and no a priori protocol was published. The review objectives, eligibility criteria and search strategy were nonetheless specified in advance and are reported here in full. A complete PRISMA-ScR checklist is provided in the Supplementary Material as File S1: PRISMA-ScR checklist.

The review addressed the following question: how are structural inequalities in ambulatory healthcare documented in the literature, how are they associated with access, quality, and patient safety, and what gaps remain in the evidence base—particularly regarding patient safety? The question was operationalised using the Population–Concept–Context (PCC) framework recommended by JBI [20]. Within this framework, the population was patients receiving or seeking ambulatory healthcare services. The concept was structural inequalities arising from socioeconomic status, geography, race or ethnicity, disability, immigration status, and institutional factors. The context was ambulatory healthcare settings across all geographic regions and health systems. In this review, patient safety was defined as freedom from preventable harm arising during ambulatory care. This encompasses diagnostic error (missed, delayed, or incorrect diagnosis), medication-safety failures (non-administration due to access barriers, adherence failures, and prescribing or dispensing errors), and breakdowns in care coordination and continuity. Throughout, a distinction is drawn between direct patient-safety events and indirect, pathway-level evidence. Direct patient-safety events are discrete instances of preventable harm arising during ambulatory care, such as diagnostic or medication errors. Indirect, pathway-level evidence refers to situations in which structural disadvantage (for example limited health literacy or unstable housing) is associated with conditions that plausibly raise the risk of harm without a safety event being measured directly. The latter is treated as hypothesis-generating rather than direct evidence of harm.

### 2.1. Search Strategy

A search strategy was developed in collaboration with an information specialist and applied to PubMed/MEDLINE and Scopus. It was structured around three concept blocks: Block A (ambulatory care terms), Block B (structural inequality terms), and Block C (outcome terms covering access, quality, and patient safety). Searches were limited to English-language publications from 2010 to 2026 and were conducted on 1 June 2026. Both Medical Subject Headings (MeSH) and free-text keywords were used for PubMed/MEDLINE, and subject headings combined with keywords were used for Scopus. Reference lists of included studies and relevant reviews were hand-searched, yielding 8 additional records. Complete search strings are provided in Appendix A. Two multidisciplinary databases with complementary coverage were used: PubMed/MEDLINE for biomedical and health-services literature and Scopus for wider coverage of social-science, health-policy, and nursing sources. This pairing is an established and pragmatic basis for a scoping review, whose purpose is to map the breadth of the evidence rather than to retrieve every record exhaustively. Specific structural dimensions such as disability, rurality, language, insurance, private-equity ownership and implicit bias were not entered as separate search terms. Instead, they were captured within the broader structural-inequality and outcome concept blocks (Blocks B and C) and applied during screening; this favoured sensitivity over specificity and avoided prematurely narrowing the multidisciplinary evidence base.

### 2.2. Eligibility Criteria

Studies were eligible if they examined ambulatory healthcare services, addressed at least one structural determinant of inequality (socioeconomic status, geography, race or ethnicity, disability, immigration status, health-system design, or institutional factors), reported outcomes relating to access, quality of care, or patient safety, and were published between 2010 and 2026. Because a scoping review maps a heterogeneous evidence base, four types of sources were eligible and were charted separately: primary empirical studies, evidence syntheses (systematic and scoping reviews), conceptual or theory-building papers and policy or economic analyses. Primary empirical studies were excluded if they were set exclusively in inpatient or residential care, examined only individual-level risk factors without structural determinants, provided insufficient methodological detail (for example, conference abstracts) or were published before 2010. Editorials and opinion pieces lacking structured data or an explicit analytical framework were excluded, whereas policy and economic analyses that presented structured evidence were retained as a distinct source type. Evidence syntheses and conceptual sources were eligible where they addressed structural mechanisms transferable to ambulatory care, including one scoping review of racism in healthcare encounters that spans inpatient and outpatient settings [24].

### 2.3. Study Selection

**Figure 1:**
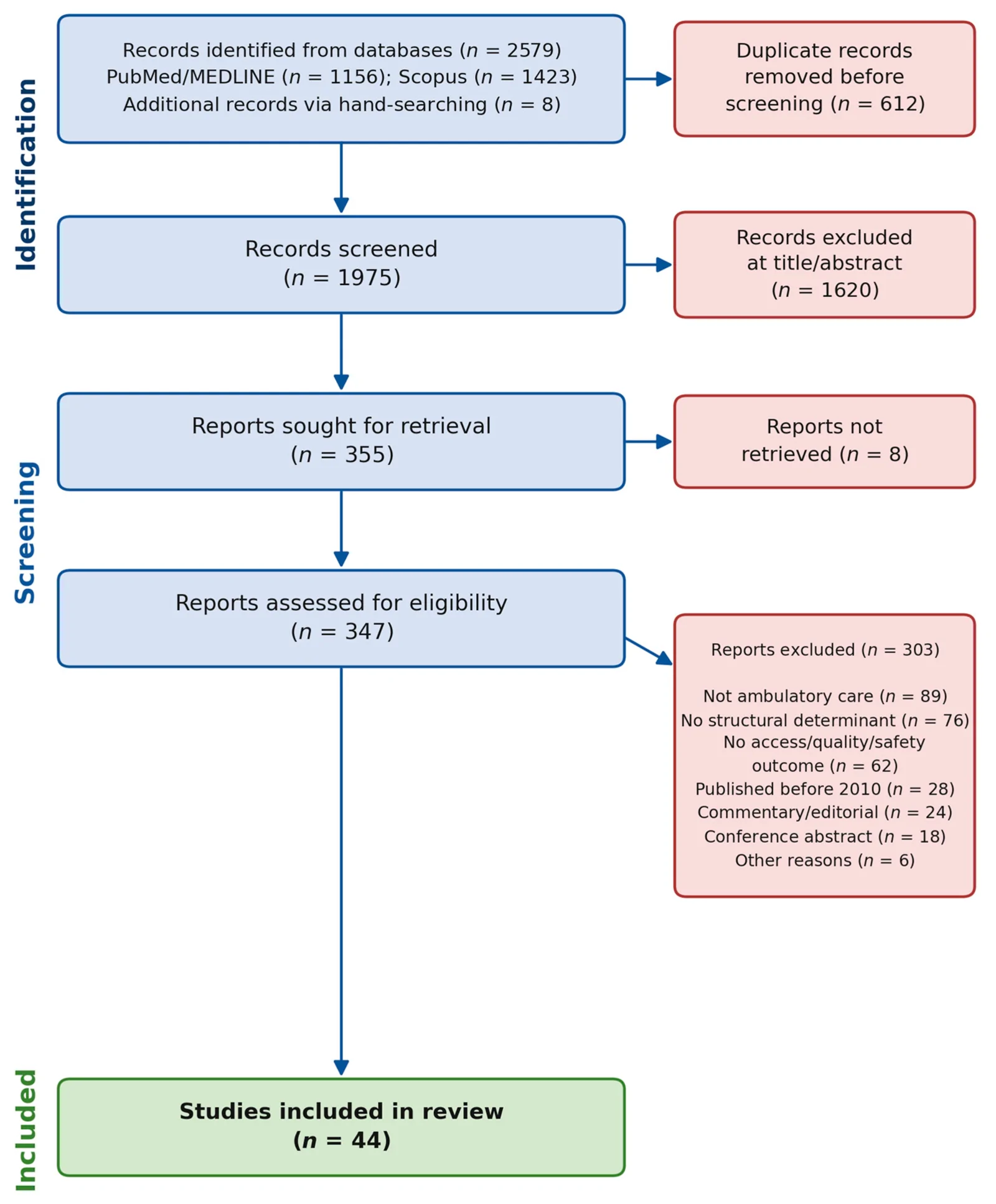
PRISMA-ScR flow digram of study identification, screening and inclusion.

A two-stage screening process was applied and documented in a PRISMA-ScR flow diagram (Figure 1). Database searching identified 1156 records in PubMed/MEDLINE and 1423 in Scopus, supplemented by eight records from hand-searching, for 2587 records in total. After removal of 612 duplicates, 1975 records were screened by title and abstract, of which 1620 were excluded. Of the 355 reports sought for retrieval, 8 could not be obtained, leaving 347 full-text articles assessed for eligibility. A further 303 reports were excluded: 89 did not address ambulatory care, 76 did not involve a structural determinant, 62 did not report access, quality, or safety outcomes, 28 were published before 2010, 24 were commentaries or editorials, 18 were conference abstracts, and 6 were excluded for other reasons. This yielded 44 studies for inclusion. Screening was performed independently by two reviewers, with disagreements resolved through discussion or adjudication.

A predefined data-extraction form was developed and piloted on five randomly selected included studies. Extracted fields comprised structural characteristics, population characteristics, healthcare context, study design and methodology, outcomes measured, key results, the mechanisms linking structural factors to outcomes, and bibliographic information. Extraction was performed independently by two reviewers, and discrepancies were resolved by consensus. In keeping with established scoping-review methodology, no formal critical appraisal of the included studies was undertaken [20,22]. Study design and methodological characteristics were nonetheless documented to support interpretation of the findings and identification of evidence gaps.

### 2.4. Data Synthesis

Extracted data were synthesised narratively across three interdependent domains: access to ambulatory care, quality of ambulatory care, and patient safety in ambulatory settings. Within each domain, findings were grouped thematically according to the structural inequality examined. Consistent with the review’s framing, the synthesis was organised to trace how structural factors in the access and quality domains propagate towards patient-safety outcomes, with particular attention to intersections between structural determinants and to differences across health-system contexts.

## 3. Results

### 3.1. Overview of Included Studies

The review included 44 studies published between 2010 and 2026. Most originated in the USA (*n* = 31), followed by international or multinational analyses spanning several countries or regions (*n* = 6), Europe (*n* = 4), Asia (*n* = 2), and Canada (*n* = 1). The evidence base was methodologically heterogeneous and comprised both primary empirical studies (cross-sectional, cohort, experimental, and quasi-experimental designs, and mixed-methods studies) and evidence syntheses or conceptual and theory-building analyses (*n* = 16); the latter were retained where they reported structured evidence or established measurement frameworks rather than opinion, consistent with the exclusion of commentaries and editorials. Because several included reviews synthesise overlapping primary studies, findings should be read as a map of the evidence landscape rather than as independent observations, and the potential for overlap is acknowledged as a limitation. Clinical and health-system foci were diverse: oncology and cancer-related care predominated (*n* = 15), with the remaining studies spanning primary and general ambulatory care, pharmacy and medicines access, health-system organisation and financing, disability- and language-related access, maternal and perinatal care, and digital or pandemic-related care. Notably, patient safety was rarely the primary outcome; where safety-relevant harms were reported they were generally described in relation to access or quality failures rather than studied directly—an imbalance that itself motivates the synthesis below. The characteristics and key findings of the included studies are charted in Table 1. By source type, the evidence base comprised 25 primary empirical studies, eight evidence syntheses (systematic or scoping reviews), eight conceptual or theory-building papers and three policy or economic analyses. Findings from each source type are reported and interpreted separately. Given the absence of formal critical appraisal, no source type was weighted above another.

### 3.2. Structural Inequalities in Access

Access was one of the two most extensively documented domains. The included studies described four recurring structural access barriers, several of which intersect with the safety concerns examined in Section 3.4.

#### 3.2.1. Geographic and Spatial Disparities

Geographic maldistribution of resources was a consistent structural barrier. Chen et al. [34] found that disparities in the allocation of primary healthcare resources accounted for more than 46% of overall inequality in Guangzhou, with population served explaining more than 72% of the inequity in resource distribution. Shaltynov et al. [56] reported moderate inequality in outpatient care in Kazakhstan, with fewer hospital beds and workers per capita in the south and west despite universal coverage. Among the Nordic countries, Finland recorded the lowest lung-cancer survival, linked to restricted primary-care access to computed tomography and the longest primary-care waiting times in the region [42]. Caldwell et al. [25] showed that rural residence was an independent disadvantage for African Americans relative to their urban counterparts, who were less likely to be screened for cholesterol (OR = 0.37; 95% CI: 0.25–0.57) and cervical cancer (OR = 0.48; 95% CI: 0.29–0.80). Delayed screening and diagnosis of this kind are recognised antecedents of the diagnostic-safety failures discussed in Section 3.4.

#### 3.2.2. Financial and Insurance-Related Barriers

Financial barriers embedded in payment systems were documented in detail. Ismail et al. [40] found that abandonment of specialty drugs rose sharply once patient cost-sharing exceeded US$100, with prior authorisation associated with treatment delays and higher discontinuation. Anderson et al. [26] reported that, across four clinical scenarios, enrollees in Medicare Advantage (the private managed-care alternative to traditional fee-for-service Medicare) were 5–16 percentage points more likely to access low-cost Part B drugs (clinician-administered outpatient drugs covered under US Medicare Part B) than traditional Medicare beneficiaries. Agarwal et al. [25] analysed 27 clinical guidelines and found that only 15% contained specific financial information, 33% addressed burden management, and 78% acknowledged financial toxicity risk. In Ireland, biosimilar adoption produced annual savings of €80 million but coincided with more limited access to novel therapies than in comparable EU countries [43]. Treatment delay and discontinuation associated with these barriers may themselves constitute medication-safety events for patients with time-sensitive conditions.

#### 3.2.3. Linguistic, Cultural and Disability-Related Barriers

Parker et al. [52] found that limited-English-proficiency Latino patients with diabetes who transferred to a language-concordant physician achieved a 10% improvement in glycaemic control (95% CI: 2–17%; *p* = 0.01) and a 9% improvement in LDL control (95% CI: 1–17%; *p* = 0.03), underscoring how communication barriers degrade disease control and, by extension, safety. Lagu et al. [47] surveyed 256 sub-specialist practices and found that 22% could not accommodate wheelchair users, rising to 44% in gynaecology. Krahn et al. [44] argued that disability should be treated as a health-disparity population, given that more than 12% of the USA population faces avoidable disadvantage requiring improved access and workforce capacity.

#### 3.2.4. Pharmacy Access as a Structural Determinant

Qato et al. [54] demonstrated that, between 2000 and 2012, segregated minority neighbourhoods in Chicago had fewer pharmacies than White or integrated neighbourhoods, with pharmacy deserts concentrated in Black neighbourhoods. Guadamuz et al. [37] confirmed that Black and Hispanic/Latino neighbourhoods continued to have fewer pharmacies and were more likely to experience closures than White neighbourhoods (14.1% and 15.9% versus 11%). Earlier distributional work by Shin et al. [65] informed this line of inquiry. Because pharmacies are the proximal site of medication dispensing, counselling, and reconciliation, their inequitable distribution is a plausible structural contributor to the medication-safety failures examined below.

### 3.3. Structural Inequalities in Quality

Quality was the second extensively documented domain, addressed by a comparable number of studies. Where access determines whether care is reached, quality concerns whether the care delivered is appropriate and equitable. The reviewed evidence identified structural quality deficits operating through clinical decision-making, industry influence, and payment design.

FitzGerald and Hurst [35] reviewed 42 studies and found evidence of implicit bias in 35, with correlational studies consistently linking bias to lower-quality care across race, gender, age, and weight. Shields et al. [57] reported that oncologists were markedly less likely to recommend opioids to a Black than to a White standardised patient (OR = 0.24; 95% CI: 0.07–0.81), whereas no such difference appeared among primary-care physicians—suggesting that specialty-specific cultures can amplify the impact of bias. Although focused on inpatient settings and therefore included only as conceptual context, Merz et al. [24] documented analogous mechanisms of structural racism that plausibly extend to ambulatory encounters. These mechanisms include differential treatment, spatial segregation of patients, and discrimination by other service users. Biassed assessment and undertreatment of these kinds are associated with diagnostic and treatment errors for the patients affected.

Carey et al. [33] found that marketing payments increased cancer drug prescribing by 4% without any corresponding mortality benefit. Pokorny et al. [53] reported that 86% of USA oncology guideline authors had received industry payments, associated with neutral or negative effects on prescribing quality. Kanter et al. [41] documented that medically integrated dispensing in oncology rose from 12.8% to 32.1% between 2010 and 2019, with higher concentrations in areas with larger Black populations (*p* < 0.001), raising equity concerns about how prescribing and dispensing incentives are distributed.

Walker et al. [61] found that the Oncology Care Model (a US Medicare bundled-payment demonstration for chemotherapy) generated drug-cost savings for prostate and lung cancer that were offset by administrative costs. Overall, novel therapy prescribing did not differ by participation, although second-line immunotherapy in lung cancer did (adjusted difference-in-differences: 17.4 percentage points; 95% CI: 4.8–30.0; *p* = 0.007). Site of care also shaped treatment: physician-office settings used erythropoiesis-stimulating agents more frequently than hospital outpatient departments (OR = 1.72; 95% CI: 1.53–1.94), indicating that reimbursement structures influence drug use independently of clinical need [48].

Mahendraratnam et al. [49] identified outpatient hospital settings as a risk factor for underuse of guideline-concordant antiemetics relative to physician offices (RR = 1.28; 95% CI: 1.25–1.30; *p* < 0.0001). Trotta et al. [60] showed that a structural policy intervention in Italy successfully shifted prescribing from branded to biosimilar filgrastim (34.4% to 49.8%), demonstrating that system-level levers can improve guideline concordance. Underuse of supportive and guideline-concordant care of this kind is a recognised antecedent of preventable harm.

### 3.4. Patient Safety: A Sparse Evidence Base

Patient safety was by far the least-studied domain: four of the 44 included studies addressed it, compared with 17 each for access and quality. Of these four, only Singh et al. [58] examined discrete safety events directly, through missed and delayed diagnoses; Berkman et al. [29] and St. Martin et al. [59] provided indirect, pathway-level evidence linking limited health literacy and maternal homelessness to conditions associated with harm, and Tsui et al. [9] contributed a conceptual framework rather than empirical harm data. The available evidence is therefore best read as illustrative of how structural inequalities in access and quality may relate to preventable harm for vulnerable patients rather than as a developed body of safety research. Singh et al. [58] found that pneumonia (6.7%), decompensated heart failure (5.7%), and acute renal failure (5.3%) were the conditions most often missed in primary care. Most diagnostic process failures occurred within the patient–practitioner encounter (78.9%), spanning history-taking (56.3%), examination (47.4%), and the ordering of diagnostic tests (57.4%). A substantial proportion carried moderate-to-severe potential for harm (43.7%). These encounter-level failures are consistent with the bias and time-pressure mechanisms identified in Section 3.3.

Health literacy emerged as a structural amplifier of medication-related harm. Berkman et al. [29] found that lower health literacy was consistently associated with poorer medication adherence, greater hospitalisation, and higher emergency-care use, contributing in part to racial disparities in outcomes. This links the access and communication barriers of Section 3.2 to downstream safety events. Extra-systemic structural determinants compounded these risks: St. Martin et al. [59] found that maternal homelessness was associated with preterm delivery across all gestational ages (adjusted ORs: 1.62–2.19) and with severe neonatal complications, including hypoxic–ischaemic encephalopathy (aOR = 14.38; 95% CI: 3.90–53.01). Taken together, these findings are consistent with a systems view in which structural pressures, rather than individual patient characteristics, are associated with conditions for unsafe ambulatory care [6,9].

### 3.5. Cross-Cutting Themes: Intersectionality, Measurement and System Context

Bowleg [31] advanced an intersectionality framework centred on the compounded disadvantage arising from intersecting social categories, while Hardeman et al. [38] examined structural racism and the role of health professionals in supporting Black lives. Krieger et al. [45] showed that the Index of Concentration at the Extremes, which combines income and racial/ethnic segregation, was more strongly associated with health outcomes than single-dimension measures (for infant mortality, low-income Black versus high-income White neighbourhoods: rate ratio 2.93; 95% CI: 2.11–4.09). Millett et al. [51] found that disproportionately Black counties accounted for 52% of USA COVID-19 cases and 58% of deaths even after adjustment for poverty and comorbidity, and Whaley et al. [62] reported lower telemedicine adoption in lower-income and majority-minority ZIP codes, perpetuating a digital divide in access.

System context further shaped these patterns. Wong et al. [64] identified facility type, medicine type, and patient socioeconomic status as determinants of access in low- and middle-income countries. Rosendahl et al. [55] found that Baltic general practitioners referred more readily than their Nordic counterparts, yet survival was lower—indicating that primary-care access alone is insufficient without secondary-care capacity. Borsa et al. [30] identified, across eight countries, a positive association between private-equity ownership and higher patient costs, with mixed or harmful effects on quality. Bender et al. [28] found that only half of Canadians with a cancer diagnosis were willing to consent to electronic storage of social-determinants data, with privacy and discrimination concerns constraining structural data collection.

### 3.6. Summary of Evidence-Gaps

The literature revealed persistent gaps: limited evidence on institutional-level interventions; predominantly USA-based evidence that constrains generalisability; scarce longitudinal tracking of how inequalities evolve; marked underrepresentation of patient safety relative to access and quality; and insufficient examination of digital-health expansion beyond the COVID-19 context. Foundational conceptual frameworks for measuring structural racism [4,36,46,63] remained only sparsely applied in ambulatory-care intervention studies. Rodriguez et al. [13] highlighted emerging digital-health policy environments, and Artiga and Hinton [27] documented expanding Medicaid initiatives to address social determinants, although implementation challenges persisted. Hu and Nerenz [39] found no significant association between 340B drug pricing (a US federal programme requiring manufacturers to supply outpatient drugs to safety-net providers at reduced prices) and expensive cancer drug use once patient and clinical factors were accounted for, suggesting that some structural pricing mechanisms may exert less influence than individual clinical characteristics.

## 4. Discussion

This scoping review identified 44 studies examining structural inequalities in ambulatory care and their effects on access, quality, and patient safety. The most striking feature of the mapping is the marked asymmetry in attention across the three domains: access and quality were each addressed by 17 studies, whereas only four examined patient safety directly. This disparity is itself a principal finding. Where the evidence does touch on safety, it is consistent with the possibility that structural inequalities operating through geographic, financial, linguistic, organisational, and institutional channels relate to preventable harm for disadvantaged patients. This extends the conceptual frameworks of Marmot and Bell [3] and Bailey et al. [4] into a domain that remains largely unexamined. Any such link should be read as a hypothesis generated by the mapping rather than a demonstrated causal pathway, given the sparse and largely indirect safety evidence.

Access was extensively documented. Geographic maldistribution emerged as a fundamental obstacle in Guangzhou [34], Kazakhstan [56], and the rural–urban USA [32], independent of insurance status. Financial barriers, namely cost-sharing [40] and insurance fragmentation [26], added further layers of disadvantage, while historical residential segregation and market forces were associated with pharmacy deserts in minority communities [37,54]. The COVID-19 pandemic intensified these inequities, with lower-income and majority-minority communities experiencing fewer in-person visits and uneven telemedicine adoption [62]. These barriers disrupt timely diagnosis and continuity of treatment, which are recognised antecedents of diagnostic and medication-safety failures.

Quality was addressed by the same number of studies as access. The reported determinants fell into three groups: payment-model incentives [50,61], provider implicit bias [35], and industry influence on prescribing [33,53]. The effect of implicit bias was not uniform across settings. Shields et al. [57] reported a larger racial disparity in pain management among oncologists than among primary-care physicians, which suggests that specialty-specific norms may modify how bias is expressed. Evidence on payment reform was mixed: the Oncology Care Model reduced drug costs but added administrative expenditure and had no consistent effect on access to novel therapies [50,61]. Financial incentives alone therefore appear insufficient to secure equitable quality.

Patient safety was the least represented domain. The available evidence indicates that patients with limited health literacy and complex social needs experience disproportionate diagnostic error, medication-safety failures, and breakdowns in care coordination [29,59]. Process failures during clinical encounters were frequent and often occurred several at once [58]. This pattern is consistent with the view that structural pressures in ambulatory delivery contribute to conditions in which harm is more likely for disadvantaged patients, and that safety is shaped principally at the level of the system rather than the individual provider [6,9]. This large and socially patterned safety deficit is corroborated by the wider primary-care and health-system literature [10,11]. Treating patient safety as an equity outcome has a direct methodological implication: safety surveillance that is not stratified by structural disadvantage will underestimate harm in the populations most exposed to it.

Interpreting these findings requires attention to intersectionality. Millett et al. [51] showed that COVID-19 disparities persisted after adjustment for poverty and comorbidity, consistent with Krieger et al. [45] in indicating that neither racial nor economic segregation alone fully accounts for the association with outcomes. The frameworks of Bowleg [31] and Krieger [46] provide analytic tools for these interactions, but the reviewed literature applied them only sparingly. Bender et al. [28] identified a tension relevant to any equity-oriented safety agenda: the data required to detect and address structural harm may themselves raise concerns about surveillance and discrimination among the communities most affected.

Most studies were conducted in the USA (*n* = 31). This concentration limits the transferability of the findings to other health systems. The smaller body of non-USA evidence indicates that several structural barriers recur under universal coverage, so these barriers are not solely artefacts of fragmented financing. Universal coverage did not eliminate structural inequality, as shown by geographic inequity in Kazakhstan [56], waiting-time disparities in Finland [42], and resource-allocation inequity in China [34]. A scoping review of quality of care under universal health coverage reached a parallel conclusion, reporting quality to be suboptimal (most markedly in low- and middle-income systems) and a global shortage of equity-sensitive quality measures [66]. Across 27 European countries with near universal coverage, self-reported unmet need for healthcare still ranged from 5.9% in Cyprus to 42.4% in Portugal, with long waiting times the leading barrier [67]. Rosendahl et al. [55] found that primary-care access alone was insufficient: cancer survival was lower in the Nordic than in the Baltic states despite higher investigation and referral rates. Borsa et al. [30] found that private-equity ownership consistently raised costs and had mixed-to-negative effects on quality across eight countries, identifying ownership structure as a structural determinant that operates largely outside clinical decision-making. Beyond this review’s systematic search, nationwide Kazakhstani data on legally registered offences against health (medical errors) offer contextual support: the recorded rate fell from 4024 to 2533 per 100,000 population between 2015 and 2019, and the authors call for a State programme to monitor medical error, underscoring that patient safety in transition economies is similarly shaped by structural and system-level conditions [68].

The findings support several policy directions. Addressing geographic maldistribution requires allocation mechanisms driven by population need rather than market signals. Reducing financial harm requires structural reform of cost-sharing and prior-authorisation processes that delay treatment. Linguistic and cultural accessibility should be embedded within quality standards rather than treated as an optional addition. Industry influence on prescribing requires greater transparency and conflict-of-interest management. Patient-safety interventions should target system vulnerabilities rather than rely on individual provider education alone. In low- and middle-income systems, where safety reporting infrastructure is weakest, strengthening reporting culture and surveillance capacity is a precondition for such targeting [69]. Priorities for future research include longitudinal designs that track how structural inequalities and policy interventions evolve; institutional-level interventions such as workforce diversification and structural-competency curricula; evidence from low- and middle-income and European systems; and examination of how artificial intelligence and algorithmic decision-making may reproduce or amplify existing disparities in ambulatory care.

## 5. Conclusions

This scoping review charted the evidence on structural inequalities in ambulatory care and their implications for access, quality, and patient safety. Across 44 studies published between 2010 and 2026, structural inequalities embedded in geographic distribution, financial barriers, exclusionary institutional cultures and market-driven organisational practices were associated with system-wide disadvantage for vulnerable populations. These findings support and extend the conceptualisations of Marmot and Bell [3], Bailey et al. [4], and Williams and Cooper [5] by specifying how institutional arrangements shape care outcomes. They also identify patient safety as the dimension in which structural-equity evidence is most clearly lacking.

Spatial, financial, linguistic, and disability-related barriers obstruct access without regard to clinical need, and market mechanisms across financing systems, from the USA to China, Kazakhstan, and the Nordic countries, fail to correct geographic disparities in infrastructure. Populations disadvantaged by race, ethnicity, socioeconomic status, or disability face additional financial barriers through cost-sharing, prior authorisation, and insurance fragmentation. Emerging structural determinants, including pharmacy deserts and digital exclusion during the COVID-19 pandemic, continue to reproduce existing inequalities.

Care quality is also shaped by structural determinants, including provider implicit bias, industry influence, payment-model incentives, and site-of-care reimbursement, that systematically channel poorer care towards disadvantaged populations. Specialty cultures can exacerbate racial disparities in pain care, and private-equity ownership generally raises costs while lowering quality. These patterns indicate a need for structural rather than individual-level solutions.

Patient safety was the least-studied domain, examined directly by only 4 of the 44 studies. The limited available evidence covers diagnostic error, medication-safety failures, and care-coordination breakdowns falling disproportionately on vulnerable populations, alongside a high frequency of encounter-level process failures. This evidence is consistent with structural barriers contributing to the conditions for harm but is too sparse to support firm conclusions. This scarcity is the principal evidence gap identified by the review. Patient safety in ambulatory care should therefore be treated as a priority equity outcome: studied directly, measured with stratification by structural disadvantage, and addressed through system-level redesign, rather than inferred indirectly from the access and quality literature.

Several gaps remain. The literature is dominated by USA evidence and generalises poorly to universal-coverage systems; longitudinal tracking of structural inequality is scarce; and institutional-level interventions, including workforce diversification, cultural-safety training, and structural-competency curricula, require further study. The intersection of artificial intelligence and digital-health expansion with structural inequality requires sustained attention to ensure that existing biases are not automated. Addressing structural inequalities will require coordinated change across levels: equitable resource distribution, reform of cost-sharing, transparency regarding industry influence, and the integration of linguistic and cultural inclusion into quality standards, alongside organisational redesign to reduce diagnostic error and workforce capacity to address social determinants of health. Equitable ambulatory care can be achieved only through comprehensive, multilevel approaches that place patient safety at the centre of the equity agenda.

## 6. Strengths and Limitations

This review has several strengths: a systematic, reproducible search developed with an information specialist; a transparent PCC framework; duplicate independent screening and extraction; and an integrative synthesis spanning access, quality, and patient safety rather than a single dimension of disadvantage. The findings are subject to several limitations. First, the search was restricted to two databases (PubMed/MEDLINE and Scopus) and to English-language publications, and grey literature was captured only through hand-searching, so relevant evidence in other databases, languages, or unpublished sources may have been missed. Second, consistent with scoping-review methodology, no formal critical appraisal was undertaken, so the included studies vary in methodological rigour and the synthesis cannot weight findings by quality. Third, the evidence base is heterogeneous and combines primary studies, evidence syntheses, and conceptual frameworks; because several reviews draw on overlapping primary studies, some findings are not statistically independent. Fourth, the evidence is concentrated in the USA, which limits transferability to universal-coverage and low- and middle-income systems. Fifth, patient safety was rarely studied directly, so the proposed pathways from inequitable access and quality to preventable harm are interpretive and hypothesis-generating rather than empirically established and require testing in purpose-designed studies.

## Figures and Tables

**Table 1:** Characteristics and key findings of the 44 included studies.

Study [Ref.]	Country	Study Design	Clinical/Health System Focus	Structural Inequality Domain	Outcome Domain(s)	Key Finding(s)
Agarwal et al. [25]	International	Systematic review	Oncology (financial burden)	Financial/Insurance	Quality	Only 15% of 27 clinical guidelines contained specific financial information; 33% specified burden management; 78% recognised financial toxicity risk.
Anderson et al. [26]	USA	Cross-sectional/comparative	Health system (Medicare)	Financial/Insurance	Access	Medicare Advantage enrollees were 5–16 percentage points more likely to access low-cost Part B drugs than traditional Medicare beneficiaries across four clinical scenarios.
Artiga & Hinton [27]	USA	Policy commentary	Medicaid/Social determinants	Social determinants/Institutional	Access	Documented growing Medicaid initiatives to address social determinants of health; implementation challenges persist across diverse system contexts.
Bailey et al. [4]	USA	Conceptual analysis/Review	General ambulatory care	Structural racism (framework)	Cross-cutting	Provided foundational conceptual framework for structural racism measurement; defined structural inequalities as systemic differences resulting from social, economic, and political organisation of society.
Bender et al. [28]	Canada	Cross-sectional survey	Oncology	Institutional/Social determinants data collection	Cross-cutting	Only half of Canadians with cancer diagnosis agreed to electronic storage of SDOH data; privacy concerns and discrimination fears negatively affected structural SDOH data collection.
Berkman et al. [29]	USA	Systematic review	General ambulatory care	Socioeconomic/Health literacy	Patient safety	Lower health literacy consistently associated with poorer medication adherence, increased hospitalizations, and emergency-care use, contributing to racial disparities.
Borsa et al. [30]	Multinational (8 countries)	Systematic review	Health system (private equity)	Organisational/Market-driven	Access, Quality	Positive link between private-equity ownership and increased costs to patients; mixed or harmful effects on quality across eight countries.
Bowleg [31]	USA	Conceptual analysis	General ambulatory care	Intersectionality framework	Cross-cutting	Proposed intersectionality framework focusing on compounded disadvantage at intersections of multiple social categories.
Caldwell et al. [32]	USA	Cross-sectional analysis	Primary care/General practice	Geographic/Rural–urban; Race/ethnicity	Access	Rural African Americans less likely to be screened for cholesterol (OR = 0.37) and cervical cancer (OR = 0.48) compared to urban counterparts; rural status independent disadvantage.
Carey et al. [33]	USA	Quasi-experimental	Oncology	Industry influence	Quality	Marketing payments led to 4% rise in cancer drug prescriptions but had no effect on mortality; industry influence on prescribing quality.
Chen et al. [34]	China (Guangzhou)	Cross-sectional analysis	Primary care	Geographic/Resource allocation	Access	Disparity in primary healthcare resource allocation accounted for >46% of overall inequality; population served accounted for >72% of inequity in resource allocation.
FitzGerald & Hurst [35]	International	Systematic review	General ambulatory care	Provider implicit bias	Quality	35 of 42 studies detected implicit bias; all correlation studies reported significant positive correlations between implicit bias and lower-quality care by race, gender, age, and weight.
Gee & Ford [36]	USA	Conceptual analysis	General ambulatory care	Structural racism (framework)	Cross-cutting	Provided foundational conceptual framework linking structural racism to health inequities; examined institutional and policy-level mechanisms.
Guadamuz et al. [37]	USA	Cross-sectional analysis	Pharmacy access	Geographic/Racial segregation	Access	Black and Hispanic/Latino neighbourhoods had fewer pharmacies than white neighbourhoods; minority neighbourhoods more likely to experience pharmacy closures (14.1% and 15.9% vs. 11%).
Hardeman et al. [38]	USA	Conceptual analysis	General ambulatory care	Structural racism	Cross-cutting	Explored structural racism and health professional roles in supporting Black lives; added conceptual structure for how structural systems worsen racial health inequities.
Hu & Nerenz [39]	USA	Observational study	Oncology (340B hospitals)	Financial/Pricing mechanisms	Quality	No significant association between 340B hospital drug pricing and expensive cancer drug use once patient and clinical factors accounted for; structural pricing less influential than individual characteristics.
Ismail et al. [40]	International	Systematic review	Specialty drugs	Financial/Cost-sharing; Prior authorization	Access	When patient cost-sharing exceeded $100, up to 75% drop in abandonment rates for specialty drugs; significant treatment delays and higher discontinuation with prior authorization.
Kanter et al. [41]	USA	Trend analysis	Oncology	Industry influence/Organisational	Quality	Medically integrated dispensing in oncology increased from 12.8% to 32.1% (2010–2019); higher percentages of Black population in areas where oncologists were also dispensing (*p* < 0.001).
Khalife et al. [42]	Nordic countries (Finland, Denmark, Norway, Sweden, Iceland)	Comparative cohort	Oncology (lung cancer)	Geographic/Resource distribution	Access, Quality	Finland had lowest lung-cancer survival due to reduced CT access in primary care and longest waiting times among Nordic countries.
Kieran et al. [43]	Ireland	Policy commentary/economic analysis	Oncology (biosimilars)	Financial/Payment systems	Access	Biosimilar integration resulted in annual savings of €80 million; however, limited access to novel therapies compared to other EU countries.
Krahn et al. [44]	USA	Conceptual analysis	Disability health disparities	Disability status	Access	Disability should be considered a health disparity; >12% of US population faces avoidable disadvantages requiring better healthcare access and workforce capacity.
Krieger et al. [45]	USA	Ecological analysis	General population health	Intersectional (income + race/ethnicity)	Cross-cutting	Index of Concentration at the Extremes (combining income and race segregation) had stronger associations with health outcomes than single-dimension measures (infant mortality rate ratio 2.93 for low-income Black vs. high-income White neighbourhoods).
Krieger [46]	USA	Conceptual analysis/Methodological	General population health	Structural measurement (framework)	Cross-cutting	Developed measures of racism, sexism, heterosexism, and gender binarism for health equity research; ecosocial analysis from structural injustice to embodied harm.
Lagu et al. [47]	USA	Cross-sectional survey	Subspecialty care access	Disability/Mobility impairment	Access	22% of 256 sub-specialist practices could not accommodate wheelchair users; gynaecology highest at 44% inability to accommodate.
Lipitz-Snyderman et al. [48]	USA	Retrospective analysis	Oncology (site-of-care)	Site-of-care reimbursement	Quality	Physician office-based care had higher rate of erythropoiesis-stimulating agent use than hospital outpatient departments (OR = 1.72; 95% CI: 1.53–1.94); site-of-care reimbursement patterns influence drug use.
Mahendraratnam et al. [49]	USA	Retrospective cohort	Oncology (antiemetics)	Site-of-care/Organisational	Quality	Outpatient hospital settings risk factor for underuse of guideline-concordant antiemetics vs. physician offices (RR = 1.28; 95% CI: 1.25–1.30; *p* < 0.0001).
Manz et al. [50]	USA	Difference-in-differences	Oncology (Oncology Care Model)	Payment model	Quality	No difference in overall novel therapy prescribing between OCM participation and non-participation; difference for second-line immunotherapy in lung cancer (adjusted DID: 17.4 pp; 95% CI: 4.8–30.0; *p* = 0.007).
Merz et al. [24]	International	Scoping review	Inpatient care (structural racism)	Structural racism	Quality	Examined structural racism in relation to differential hospitalisation rates, spatial segregation of patients, and discrimination from other health service users.
Millett et al. [51]	USA	Ecological analysis	COVID-19	Structural racism/Intersectional	Cross-cutting	Nationally disproportionately Black counties had 52% of COVID-19 cases and 58% of deaths, even after controlling for poverty and comorbidities.
Parker et al. [52]	USA	Retrospective cohort	Primary care (diabetes)	Linguistic/Language concordance	Quality	Limited-English-proficiency Latino patients transitioning to language-concordant physician had 10% improvement in glycaemic control (95% CI: 2–17%, *p* = 0.01) and 9% improvement in LDL control (95% CI: 1–17%, *p* = 0.03).
Pokorny et al. [53]	USA	Systematic review	Oncology/Haematology	Industry influence	Quality	86% of oncology guideline authors in USA had received industry payments; payments connected to neutral or negative effects on prescribing quality.
Qato et al. [54]	USA	Cross-sectional analysis	Pharmacy access	Geographic/Racial segregation	Access	Fewer pharmacies in segregated minority neighbourhoods vs. white/integrated neighbourhoods (2000–2012); significantly more pharmacy deserts in Black neighbourhoods.
Rodriguez et al. [13]	USA	Policy commentary	Digital health	Digital exclusion	Access	Highlighted emerging digital-health policy environments; telemedicine adoption less in lower-income and majority-minority zip codes, perpetuating digital divide.
Rosendahl et al. [55]	Baltic and Nordic countries	Vignette study	Primary care (cancer referral)	Geographic/Health system capacity	Access, Quality	Baltic GPs more likely to refer than Nordic counterparts; however, survival was lower, suggesting primary-care access alone insufficient without secondary care capacity.
Shaltynov et al. [56]	Kazakhstan	Spatiotemporal analysis	Health system (outpatient care)	Geographic/Resource distribution	Access	Medium inequality in outpatient care; south and west had fewer hospital beds and workers per capita despite universal health coverage.
Shields et al. [57]	USA	Randomised field experiment	Oncology (lung cancer pain management)	Provider implicit bias/Race	Quality	Oncologists significantly less likely to prescribe opioids to Black standardised patient than White standardised patient (OR = 0.24; 95% CI: 0.07–0.81); no racial difference in primary care physicians.
Singh et al. [58]	USA	Retrospective analysis	Primary care (diagnostic errors)	System-level/Process failures	Patient safety	Pneumonia (6.7%), decompensated heart failure (5.7%), acute renal failure (5.3%) highest prevalence of missed diagnoses; 78.9% process failures in patient–practitioner encounter.
St. Martin et al. [59]	USA	Retrospective cohort	Maternal/perinatal health	Social determinants/Homelessness	Patient safety	Maternal homelessness associated with pre-term delivery at all gestations (aORs: 1.62–2.19) and severe neonatal complications (hypoxic–ischemic encephalopathy aOR = 14.38; 95% CI: 3.90–53.01).
Trotta et al. [60]	Italy (Lazio Region)	Population-based utilisation study	Oncology (G-CSF prescribing)	Policy/Guideline implementation	Quality	Pharmaceutical policy intervention successfully shifted prescribing from branded to biosimilar filgrastim (34.4% to 49.8%).
Tsuei et al. [9]	USA	Conceptual framework	General ambulatory care	Systems-based health equity	Patient safety	Proposed systems-based framework for integrating health equity and patient safety; vulnerable populations disproportionately impacted by diagnostic errors, medication failures, and care-coordination failures.
Walker et al. [61]	USA	Quasi-experimental evaluation	Oncology (Oncology Care Model)	Payment model	Quality	Savings in drug costs for prostate and lung cancer in OCM, but offset by programme administration costs; no difference in overall novel therapy prescribing.
Whaley et al. [62]	USA	Cross-sectional analysis	Telemedicine/COVID-19	Digital exclusion/Socioeconomic	Access	Telemedicine less adopted in lower-income and majority-minority zip codes; perpetuated digital divide and access inequities during COVID-19.
Williams et al. [63]	USA	Review/Conceptual analysis	General ambulatory care	Structural racism (framework)	Cross-cutting	Reviewed evidence on racism and health; identified needed research directions for understanding structural determinants of health inequities.
Wong et al. [64]	Low- and middle-income countries (systematic review)	Systematic review	Non-communicable disease medicines	Socioeconomic/Facility type	Access	Factors affecting access include type of facility, medicine type, and socioeconomic status of patients in LMICs.

## Data Availability

No new data were created or analysed in this study. Data sharing is not applicable to this article.

## Supplementary Materials

The following supporting information can be downloaded at: https://www.mdpi.com/article/10.3390/ijerph23081021/s1, File S1: PRISMA-ScR checklist [21].

## Abbreviations

The following abbreviations are used in this manuscript:

AHRQ	Agency for Healthcare Research and Quality
aOR	Adjusted odds ratio
CI	Confidence interval
CT	Computed tomography
JBI	Joanna Briggs Institute
LMIC	Low-and middle-income country
MeSH	Medical Subject Headings
OECD	Organisation for Economic Co-Operation and Development
OR	odds ratio
PCC	Polulation, Concept, Context
PRISMA-ScR	Preferred Reporting Items for Systematic Reviews and Meta-Analyses extension for Scoping Reviews
RR	Risk Ratio
SDOH	Social determinants of health

## Appendix A

### Appendix A.1

Search conducted: 1 June 2026. Database: PubMed/MEDLINE. Language: English. Date range: 2010–2026.

Block A (ambulatory care): (“Ambulatory Care”[MeSH] OR “Outpatient Clinics, Hospital”[MeSH] OR “Primary Health Care”[MeSH] OR “Community Health Services”[MeSH] OR “Emergency Service, Hospital”[MeSH] OR “ambulatory care”[tiab] OR “outpatient care”[tiab] OR “outpatient services”[tiab] OR “primary care”[tiab] OR “primary health care”[tiab] OR “community health”[tiab] OR “emergency department”[tiab] OR “emergency room”[tiab] OR “walk-in clinic”[tiab] OR “urgent care”[tiab])

AND Block B (structural inequality): (“Health Equity”[MeSH] OR “Healthcare Disparities”[MeSH] OR “Social Determinants of Health”[MeSH] OR “Socioeconomic Factors”[MeSH] OR “Health Status Disparities”[MeSH] OR “structural inequality”[tiab] OR “structural inequalities”[tiab] OR “structural racism”[tiab] OR “systemic racism”[tiab] OR “institutional racism”[tiab] OR “health disparities”[tiab] OR “healthcare disparities”[tiab] OR “health inequality”[tiab] OR “health inequalities”[tiab] OR “social determinants”[tiab] OR “socioeconomic status”[tiab] OR “health equity”[tiab] OR “health inequity”[tiab] OR “health inequities”[tiab])

AND Block C (outcomes): (“Health Services Accessibility”[MeSH] OR “Quality of Health Care”[MeSH] OR “Patient Safety”[MeSH] OR “healthcare access”[tiab] OR “access to care”[tiab] OR “care access”[tiab] OR “barriers to care”[tiab] OR “quality of care”[tiab] OR “care quality”[tiab] OR “patient safety”[tiab] OR “medical error”[tiab] OR “diagnostic error”[tiab] OR “medication error”[tiab] OR “adverse event”[tiab] OR “preventable harm”[tiab]). Filters: English; 1 January 2010–31 December 2026.

### Appendix A.2

Search conducted: 1 June 2026. Database: Scopus. Language: English. Date range: 2010–2026.

TITLE-ABS-KEY(“ambulatory care” OR “outpatient care” OR “outpatient services” OR “primary care” OR “primary health care” OR “community health” OR “emergency department” OR “emergency room” OR “walk-in clinic” OR “urgent care”) AND TITLE-ABS-KEY(“structural inequality” OR “structural inequalities” OR “structural racism” OR “systemic racism” OR “institutional racism” OR “health disparities” OR “healthcare disparities” OR “health inequality” OR “health inequalities” OR “social determinants” OR “socioeconomic status” OR “health equity” OR “health inequity” OR “health inequities”) AND TITLE-ABS-KEY(“healthcare access” OR “access to care” OR “care access” OR “barriers to care” OR “quality of care” OR “care quality” OR “patient safety” OR “medical error” OR “diagnostic error” OR “medication error” OR “adverse event” OR “preventable harm”). Filters: English; article, review, article in press; 2010–2026.
