# Supplementary material for: Structural Inequalities in Ambulatory Care: A Scoping Review of Access and Quality and the Neglected Dimension of Patient Safety
Source: Int J Environ Res Public Health. 2026 Aug 4;23(8):1021. doi: 10.3390/ijerph23081021 (PMC13512547; doi:10.3390/ijerph23081021)
Supplement: Supplementary file 1 [file ijerph-23-01021-s001.zip › ijerph-4439729-supplementary.pdf]

File S1: PRISMA-ScR Checklist

Preferred Reporting Items for Systematic reviews and Meta-Analyses extension for Scoping Reviews (PRISMA-ScR): Checklist

Manuscript: Structural Inequalities in Ambulatory Care: A Scoping Review of Access and Quality and the Neglected Dimension of Patient Safety.

Note. Item wording is condensed from the official PRISMA-ScR checklist (Tricco AC, Lillie E, Zarin W, et al. PRISMA Extension for Scoping Reviews [PRISMA-ScR]: Checklist and Explanation. Ann Intern Med. 2018;169:467–473. doi:10.7326/M18-0850). Items 12 and 16 (critical appraisal) are optional for scoping reviews; a formal critical appraisal was not conducted in this review, consistent with scoping-review methodology. Locations refer to the corresponding sections of the revised manuscript.

Section	Item	PRISMA-ScR Checklist Item	Reported (location in manuscript)
**TITLE**			
**Title**	1	Identify the report as a scoping review.	Title page
**ABSTRACT**			
**Structured summary**	2	Provide a structured summary that includes (as applicable) background, objectives, eligibility criteria, sources of evidence, charting methods, results, and conclusions that relate to the review questions and objectives.	Abstract
**INTRODUCTION**			
**Rationale**	3	Describe the rationale for the review in the context of what is already known. Explain why the review questions/objectives lend themselves to a scoping review approach.	1. Introduction
**Objectives**	4	Provide an explicit statement of the questions and objectives being addressed with reference to their key elements (e.g., population or participants, concepts, and context) or other relevant key elements used to conceptualize the review questions and/or objectives.	1. Introduction; Research questions, end of Introduction / start of 2. Methods
**METHODS**			
**Protocol and registration**	5	Indicate whether a review protocol exists; state if and where it can be accessed (e.g., a Web address); and if available, provide registration information, including the registration number.	2. Materials and Methods: the review was not registered and no a priori protocol was published; objectives, eligibility criteria, and search strategy were specified in advance and are reported in full.
**Eligibility criteria**	6	Specify characteristics of the sources of evidence used as eligibility criteria (e.g., years considered, language, and publication status), and provide a rationale.	2.2 Eligibility Criteria: four source types charted separately-primary studies, evidence syntheses, conceptual papers, and policy/economic analyses.
**Information sources**	7	Describe all information sources in the search (e.g., databases with dates of coverage and contact with authors to identify additional sources), as well as the date the most recent search was executed.	2.1 Search Strategy: PubMed/MEDLINE and Scopus; hand-searching of reference lists; search conducted 1 June 2026.
**Search**	8	Present the full electronic search strategy for at least 1 database, including any limits used, such that it could be repeated.	2.1 Search Strategy; full search strings in Appendix A.
**Selection of sources of evidence**	9	State the process for selecting sources of evidence (i.e., screening and eligibility) included in the scoping review.	2.3 Study Selection; PRISMA-ScR flow diagram (Figure 1).
**Data charting process**	10	Describe the methods of charting data from the included sources of evidence (e.g., calibrated forms or forms that have been tested by the team before their use, and whether data charting was done independently or in duplicate) and any processes for obtaining and confirming data from investigators.	2.3 / 2.4 Methods: standardized charting form; extracted variables listed.
**Data items**	11	List and define all variables for which data were sought and any assumptions and simplifications made.	2.4 Data Synthesis and Table 1 column definitions.
Critical appraisal of individual sources of evidence	*12*	*If done, provide a rationale for conducting a critical appraisal of included sources of evidence; describe the methods used and how this information was used in any*	*Not performed. Consistent with scoping-review methodology; stated in Methods and in Strengths and Limitations.*

		data synthesis (if appropriate). [Optional]	
**Synthesis of results**	13	Describe the methods of handling and summarizing the data that were charted.	2.4 Data Synthesis: narrative synthesis across access, quality, and patient safety; findings reported and interpreted separately by source type.
**RESULTS**			
**Selection of sources of evidence**	14	Give numbers of sources of evidence screened, assessed for eligibility, and included in the review, with reasons for exclusions at each stage, ideally using a flow diagram.	3.1 Overview of Included Studies; Figure 1 (PRISMA-ScR flow diagram).
**Characteristics of sources of evidence**	15	For each source of evidence, present characteristics for which data were charted and provide the citations.	3.1 Overview and Table 1.
Critical appraisal within sources of evidence	*16*	*If done, present data on critical appraisal of included sources of evidence (see item 12). [Optional]*	*Not applicable (critical appraisal not conducted; see item 12). Stated in Strengths and Limitations.*
**Results of individual sources of evidence**	17	For each included source of evidence, present the relevant data that were charted and that relate to the review questions and objectives.	Table 1 and 3.2–3.4 domain results.
**Synthesis of results**	18	Summarize and/or present the charting results as they relate to the review questions and objectives.	3.1–3.6 Results, including source-type counts (25 primary studies, 8 evidence syntheses, 8 conceptual papers, 3 policy/economic analyses) and the direct-vs-indirect patient-safety distinction.
**DISCUSSION**			
**Summary of evidence**	19	Summarize the main results (including an overview of concepts, themes, and types of evidence available), link to the review questions and objectives, and consider the relevance to key groups.	4. Discussion.
**Limitations**	20	Discuss the limitations of the scoping review process.	6. Strengths and Limitations: two-database search, absence of formal critical appraisal, overlap among reviews, USA concentration, hypothesis-generating safety evidence.
**Conclusions**	21	Provide a general interpretation of the results with respect to the review questions and objectives, as well as potential implications and/or next steps.	5. Conclusions.
**FUNDING**			
**Funding**	22	Describe sources of funding for the included sources of evidence, as well as sources of funding for the scoping review. Describe the role of the funders of the scoping review.	Funding statement: no funding received.

Reference: Tricco AC, Lillie E, Zarin W, O'Brien KK, Colquhoun H, Levac D, et al. PRISMA Extension for Scoping Reviews (PRISMA-ScR): Checklist and Explanation. Ann Intern Med. 2018;169(7):467–473.
